# Infantile Constipation

**Published:** 1881-01

**Authors:** 


*L'Union Medicale* gives the following formulæ:

Acute-Scrofulous Mixture—

R.—Kali Iodid .................. ʒ ss<br>

Tr. Iodin .................. M xv<br>

Tannin .................. grs. xv<br>

Syr. Cinchona ............ ℥ i ʒ v<br>

Gum Sulep ............ ℥ v ʒ viii

Ms,—℥ i. every two hours to scrofulous adults; children proportionately.

---

Dobell's Purgative Tincture—

R.—Res. Podophyllin .......... grs. ii<br>

Essence Zingiber .......... ʒ i. M xv<br>

Spts. Vin. Recti .................. ʒ ii

Ms.—ʒi. at night when lying down, every two or three nights. Podophyllin is claimed to act mildly.

Infantile Constipation.—At a clinical lecture held at the College of Physicians and Surgeons, New York, Prof. Jacobi called attention to a form of infantile constipation not mentioned in the books. In this affection the color of the fæces is about normal, but they are deficient in moisture. They are dry and somewhat friable. The passages of young babies are never normally like this. There is evidently here a lack of moisture which may possibly arise from an insufficient secretion on the part of the intestinal glands. It may, however, arise from other causes, one of which is a peculiar anatomical condition occasionally existing in the bowels of the newborn or young infants. A few anatomists have recognized that the intestinal tract is different in the young from what it is in the old. The colon is very much larger and longer, in proportion, in the child than in the adult, and this peculiar condition often remains up to the age of five or six years. The child may have two or even three sigmoid flexures, or the real sigmoid flexure may not be found on the left side, but on the right. In the passage of the young, where the peristaltic action of the bowel is normal and the colon of the usual proportion, the fæces will not be dried out; but where the flexure is long, or there are two or three of them, the fæces will dry out. In the fœtus and the new-born the secretions of the intestines are very copious. There is a great deal of mucous and epithelium, which may become very dry and compressed—to such an amount, indeed, as to constitute actual obstruction. Dr. Jacobi stated that he has met with a number of cases in children, that could not be explained in any other way than by the supposition that there were two or three sigmoid flexures, one on top of the other, and impeding the free passage of the fæces. In the treatment of a case where such a state of things is suspected, the diet must be regulated so that there may be an abundance of water in the food. In the choice of food, oatmeal is to be given in preference to tapioca, farina, or even barley. Purgatives must not be given except in urgent cases. Injections are very useful, and cannot be dispensed with. Another cause of constipation like this may be that there is an insufficient physiological action of the muscular layer of the intestine. This may occur in feeble children. In another class of children this constipation does not appear until some six months to one year after birth, and then, from being perfectly regular, they become obstinately constipated. In this class the muscles of voluntary motion, as well as those of the intestine, become diminished in power; they are rachitic children.—*Medical and Surgical Reporter.*

